# Substitution of cysteines in the yeast viral killer toxin K1 precursor reveals novel insights in heterodimer formation and immunity

**DOI:** 10.1038/s41598-019-49621-z

**Published:** 2019-09-11

**Authors:** Stefanie Gier, Matthias Lermen, Manfred J. Schmitt, Frank Breinig

**Affiliations:** 1 Molecular and Cell Biology, Saarland University, 66123 Saarbrücken, Germany; 2 Center of Human and Molecular Biology (ZHMB), Saarland University, 66123 Saarbrücken, Germany

**Keywords:** Antifungal agents, Cellular microbiology

## Abstract

The killer toxin K1 is a virally encoded fungal A/B toxin acting by disrupting plasma membrane integrity. The connection of α and β constitutes a critical feature for toxin biology and for decades the formation of three disulphide bonds linking the major toxin subunits was accepted as status quo. Due to the absence of experimental evidence, the involvement of each cysteine in heterodimer formation, K1 lethality and immunity was systematically analysed. Substitution of any cysteine in α led to a complete loss of toxin dimer secretion and toxicity, whereas K1 toxin derivatives carrying mutations of C248, C312 or the double mutation C248-312 were active against spheroplasted cells. Importantly, substitution of the C95 and C107 in the toxin precursor completely abolished the mediation of functional immunity. In contrast, K1 toxicity, i.e. its ionophoric effect, does not depend on the cysteine residues at all. In contrast to the literature, our data imply the formation of a single disulphide bond involving C92 in α and C239 in β. This finding not only refines the current model stated for decades but also provides new opportunities to elucidate the mechanisms underlying K1 toxicity and immunity at the molecular level.

## Introduction

The secretion of proteinaceous compounds is a conserved evolutionary phenomenon and a wide-spread biological feature. Although best described for mainly bacterial toxin producers such as *Vibrio cholerae* and *Clostridium botulinum*, yeast cells are also able to secrete toxic proteins into their extracellular environment. Studies on the secretion of these killer toxins contributed significantly to the understanding of the secretory pathway as well as the processes of protein maturation and secretion in eukaryotic cells^1,2^.

*Saccharomyces cerevisiae*, the killer phenotype has meanwhile been discovered in various yeast genera contributing vastly to the structure of yeast communities^3–5^. In case of the four identified killer toxins for *S*. *cerevisiae* (K1, K2, K28, and Klus), killer strains are infected with two different double-stranded RNA viruses, M and L-A, that are persistently present in the cytoplasm of the host cell. The lethal effect of these virally encoded toxins relies on a two-staged receptor-mediated process killing sensitive yeast strains of the same or other genera. After initial binding to the respective primary receptor within the cell wall, the killer toxin is translocated to the plasma membrane level where the interaction with its secondary receptors takes place, eventually triggering the lethal effect^6^.

In case of K1, the toxin firstly binds to its primary receptor β-1,6-glucan in the yeast cell wall and is then transferred to the plasma membrane, where it interacts with Kre1p, an O-glycosylated and GPI-anchored protein^7,8^. Subsequently, the formation of cation-selective pores leads to an uncontrolled influx of protons coupled with an efflux of potassium ions^9,10^. Despite many efforts, the exact molecular mechanisms of this ionophoric effect and potential involvement of accessory proteins could not be clarified yet. Interestingly, killer yeast necessitate a particular self-immunity system against their own toxin as they possess the same receptors on their cell surface and are in principle as susceptible to their toxin as sensitive cells. This functional immunity mechanism is specific for each toxin as killer yeast display no cross-immunity when exposed to other killer toxins^11^. In case of K1, preceding studies could exclude the involvement of its secondary receptor Kre1p and the potassium channel Tok1p^8,12^. However, expression of the toxin precursor in a sensitive cell is sufficient to confer immunity to externally applied K1, clearly pointing to a mechanism involving proteins of the secretory pathway and the precursor itself^12,13^. Moreover, it has been shown that the ER-import of a construct consisting of the toxic α subunit extended with at least 31 amino acids of the γ subunit is sufficient to mediate complete immunity to previous sensitive cells^14^. In contrast to K28, where immunity is solely based on the post-translational import of the toxin precursor^15^, the exact mechanism for K1 killer toxin needs further experimental validation.

Although K28 and K1 possess a completely different mode of action, their arrangement, as well as maturation process in the secretory pathway, is comparable to classical AB toxins. Besides an N-terminally located signal sequence necessary for ER import and a pro-region with so far unknown function/s, the unprocessed precursor (preprotoxin, pptox) consists of the two major toxin subunits α and β, initially separated by a γ component. After ER import and cleavage of the signal peptide, the γ subunit is N-glycosylated, and the major toxin subunits are covalently linked via disulphide bonds^16,17^. As the K28 toxin precursor displays only one single cysteine in the toxic α subunit, only one intermolecular disulphide bond can be formed. Consequently, three out of four cysteines in β are not involved in the linkage of the major toxin subunits. Recent findings clearly illustrate the significance of the remaining sulphuric residues in the pH-dependent killing process of the toxin^18^. After recognition of the toxin’s HDEL-motif by the yeast HDEL-receptor Erd2p, the toxin dimer is internalised, transported to the ER, and eventually translocated to the cytoplasm where the α subunit induces a cell cycle arrest in sensitive yeast cells^17,19^. During the passage of the secretory pathway, the toxin molecule takes advantage of the physiological intracellular pH gradient. In the more neutral pH of the ER, thiol residues in the β subunit are deprotonated and thereby able to attack one of the remaining sulfuric residues in a nucleophilic fashion. This process results in a rearrangement of the disulphide bonds leading to the separation of the toxin subunits and the release of α into the cytoplasm. K1, in contrast, possesses six cysteine residues in total, three located in α (C92, C95, C107) and three in β (C239, C248, C312) allowing in principle the formation of three disulphide bonds (see Suplementary Fig. S1a). Consequently, the current model postulates the pairing of C92-C248, C95-C312, and C107-C239 by sequence similarity; however, experimental evidence for this assumption is still lacking.

**Figure 1:**
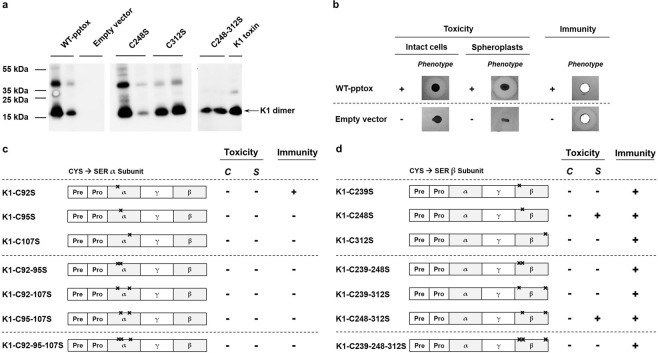
(**a**) Secretion and biological activity of K1 heterodimer are hampered by cysteine substitution. Toxin secretion was analysed via Western analysis using a polyclonal K1 antibody detecting the K1 heterodimer. Supernatant of wild-type K1 pptox (WT-pptox) and empty vector-transformed yeast, as well as K1 toxin concentrate, were used as controls indicating specific bands of approx. 18 kDa representing the mature heterodimer. Distinct bands could be observed for β single mutants C248S and C312S, and the double mutant C238-312S. No secretion of heterodimeric K1 toxin could be observed for any of the α mutated transformants. Displayed are the results of representative transformants; for improved visualisation blot images were cropped, and background brightness was adjusted. Unmodified full-length blots are presented in Supplementary Fig. S2. (**b**) Expression of wild-type K1 precursor leads to secretion of biological active toxin and the mediation of functional immunity. Wild-type K1 pptox and the empty vector were transformed in BY4742 cells, and biological activity of secreted toxin against intact and spheroplasted cells was analysed via MBA diffusion assay. Therefore, 10^6^ (intact cells) cells or approx. 10^7^ spheroplasts of the sensitive strain BY4742 were embedded into appropriate MBA media, and colonies of the transformants were streaked onto the agar plates. Biological activity was indicated by the formation of killing zones around the spotted transformants (indicated with+). Functional immunity was characterised by embedding 10^6^ cells of each transformant into ura-galactose MBA (pH 4.7) and applying 1,000 AU K1 toxin concentrate. Loss of immunity is indicated by a zone of growth inhibition and labelled with (−), whereas immunity is marked with (+). Summary of observed phenotypes for cysteine mutants in the α (**c**) and β subunit (**d**). Substitutions of cysteines with serine residues are indicated by a black cross. Tables summarise the biological activity of the cysteine-mutated K1 derivatives against intact (*C*) and spheroplasted cells (*S*) as well as the formation of functional immunity. Wild-type phenotype of the mutated K1 pptox derivatives is indicated by (+), whereas no toxicity and loss of immunity are labelled with (−).

This study aimed to identify the disulphide bond arrangement by using appropriate derivatives of the K1 precursor molecule carrying single, double, and triple cysteine mutations in either α and/or β, respectively. Furthermore, α-derivatives were utilised to determine the involvement of the cysteine residues in the toxin’s lethal mechanism. Whereas toxicity was not affected at all by any cysteine mutation in α, other combinations prevented dimer formation and/or secretion or toxicity against whole cells and spheroplasts. Remarkably, substitution of cysteines C95 and C107 in the toxin precursor led to a complete loss of immunity, implying a critical involvement of these residues within the so far unknown mechanism conferring immunity against K1. In sum, we propose the formation of only one disulphide bond linking the major toxin subunits involving C92 and C239, thereby refining the model of K1 dimer formation and function/s of its subunits stated in the literature for decades.

## Results and Discussion

Yeast killer strains possess the ability to kill other sensitive yeast cells of the same or other genera without direct cell-cell interaction. However, in contrast to bacterial toxin producers, killer yeast need to establish a specific mechanism ensuring functional immunity against their own toxin. Among the virally encoded killer toxins, K1 and K28 of the baker’s yeast *S*. *cerevisiae* belong to the best-studied proteins^20^. As classical A/B toxins, the mature heterodimers consist of one α and β subunit, respectively, covalently linked via at least one disulphide bond^21^.

In contrast to K28 and hitherto accepted in the literature, the α and β subunit of K1 toxin could potentially be linked by a maximum of three disulphide bonds (see Supplementary Fig. S1a) adding more complexity to potential rearrangement events of the mature toxin during the killing process, e. g. the embedding in the plasma membrane. The toxic effect of K1 is attributed to an energy-dependent disruption of the plasma membrane integrity and the proton transmembrane gradient, eventually cumulating in electrochemical and energetical drainage of sensitive target cells. Time-dependent analysis of transcriptome changes in sensitive cells supported previous observations showing an approximately one-hour lag phase between toxin application and the beginning of a detectable decrease in cell viability^22^. This clearly points two a more complex mechanism of K1 toxin action as well as to the potential of sensitive cells to, at least initially, combat the pore formation.

Due to incomplete knowledge of the precise killing mechanism on a molecular level, it is not known yet if β is still attached to α during pore formation or if single toxin heterodimers or even α-monomers can enter the target cell and exert intracellular effects supporting or accelerating the lethal mechanism. In a novel approach, this study thereby focused on the importance of the cysteine residues in both major toxin subunits and their potential involvement in the K1 killer toxin biology. This is of particular interest, as the observed ionophoric action of the toxin is attributed to the formation of cation-selective channels by the toxic α subunit, which possesses two highly hydrophobic α-helical regions facilitating the insertion of the subunit into lipid bilayers^23^. These specific hydrophobic sites are located between the amino acids (aa) 72–91 as well as 112–117 flanking a smaller hydrophilic region (aa 92–11). As additionally visualised via hydropathicity plot, all cysteine residues of the α subunit are positioned in this hydrophilic patch near the hydrophobic sites^16,24^ (see Supplementary Fig. S1b).

### Mutations in cysteines impede dimer secretion and biological activity

In first experiments, cysteine-mutated K1 pptox derivatives bearing single, double or triple substitutions to serine were constructed for each major subunit and expressed in the sensitive *S*. *cerevisiae* strain BY4742. Potentially resulting suicidal phenotypes were monitored under inducing conditions by serial dilution assays, and no changes in cell growth could be observed (data not shown). Secretion of K1 heterodimers was examined via non-reducing SDS-PAGE followed by Western analysis. In the control samples (K1 toxin concentrate as well as cells transformed with wild-type (WT) K1 pptox), toxin-specific signals were detected around 18 kDa. Comparable signals could only be observed for the single mutants C248S, C312S, and the double mutant C248-312S, whereas no signals could be monitored for any α mutated derivative (Fig. 1a).

Furthermore, MBA (methylene blue agar) diffusion assays were conducted to determine the biological activity of secreted K1 toxin. The sensitive strain BY4742 was embedded into the agar, and cells of each transformant were spotted onto the plates. Plasmidal expression of wild-type K1 resulted in secretion of functional toxin killing both, intact and spheroplasted cells. In contrast, empty vector-transformed cells were not able to kill sensitive yeast and were killed by extracellular K1 application (Fig. 1b). Although K1 dimer secretion could be verified for three β-mutated pptox derivatives, none of the mutants secreted K1 toxin with biological activity against intact (whole) cells. This indicates conformational changes of the toxin subunits caused by the cysteine substitutions, which potentially interfere with K1 binding to its primary receptor, the β-1,6 glucan fraction of the yeast cell wall. In order to test this assumption, toxicity against spheroplasted cells was checked. After enzymatical removement of the cell wall, toxin activity could be verified for transformants carrying the single mutation C248S, and the double mutant C248-312S (Fig. 1c,d).

Based on the results of the Western analysis, we propose the formation of only one disulphide bond involving cysteine C239 in β as this single mutant was the only β-mutated derivative defective in K1 heterodimer secretion. Simultaneously, our findings show a distinct loss of K1 secretion and toxicity as soon as any cysteine in α was substituted. This observation is in line with a previous study where a comprehensive mutational analysis of the K1 precursor was conducted; several amino acids substitutions in the α subunit abolished the ability of the toxin to kill either intact or spheroplasted sensitive cells^24^. The substitutions could negatively influence specific secondary structures in α eventually resulting in a loss of function and, consequently, the ability to form pores into lipid bilayers.

### Cysteine residues are not involved in K1 toxicity

**Figure 2:**
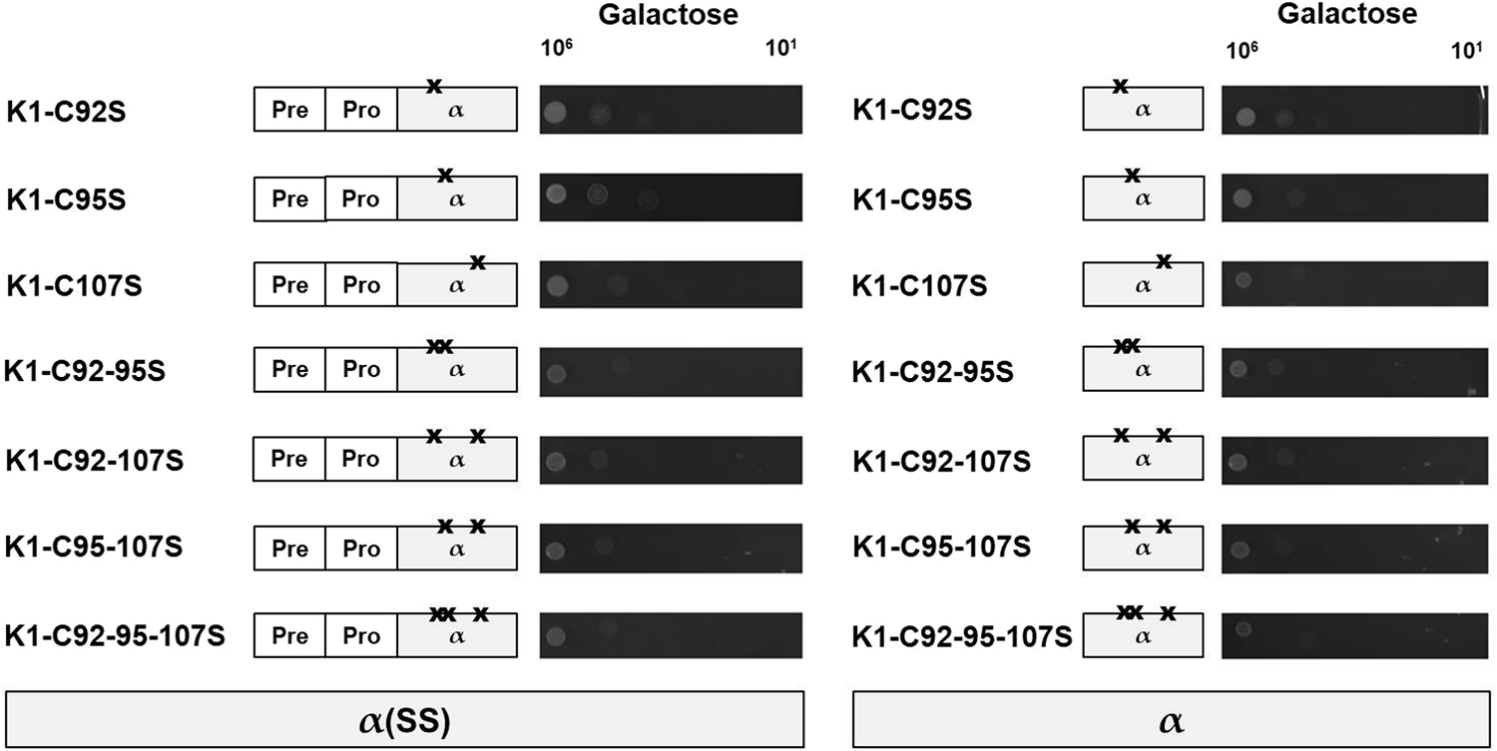
Induction of suicidal phenotype is independent of cysteine arrangement. Yeast strain *S*. *cerevisiae* BY4742 was transformed with the indicated K1 lethal constructs with (α(SS), left) or without the prepro region (α, right), and 10^6^ to 10^1^ cells were spotted onto agar plates containing galactose (induced conditions). Growth was analysed after 3 d at 30 °C, and the results of one representative experiment are displayed (n = 5).

So far, investigating possible effects of amino acid substitutions in K1 on toxicity was hampered by diminished or even blocked secretion of mutated toxin variants. In a recent study, we were able to show the ability of intracellularly expressed K1α derivatives to mimic the lethal effect of extracellularly applied toxin allowing to test K1 toxicity without the presence of β and, thus, without the need of heterodimeric toxin to be secreted^12^. Potential influences of the cysteines in the toxic effect were analysed by constructing cysteine-substituted K1α derivatives either with [α(SS)] or without the prepro-region (α). After expression of the respective constructs in the sensitive strain BY4742, transformants showed no growth impairment under non-inducing conditions (glucose, data not shown), whereas the expression of all mutated derivatives resulted in a distinctive suicidal phenotype indicated by the substantial decrease in cell growth on galactose (Fig. 2). Interestingly, as already shown for the wild-type lethal constructs, no significant difference between α and α(SS) could be observed. Our findings suggest that the insertion of the K1 α subunit in cellular membranes, i.e. the toxic effect, is not altered by cysteine mutations and thus not dependent on the presence of cysteines at all.

### C95 and 107 are important for immunity against K1

**Figure 3:**
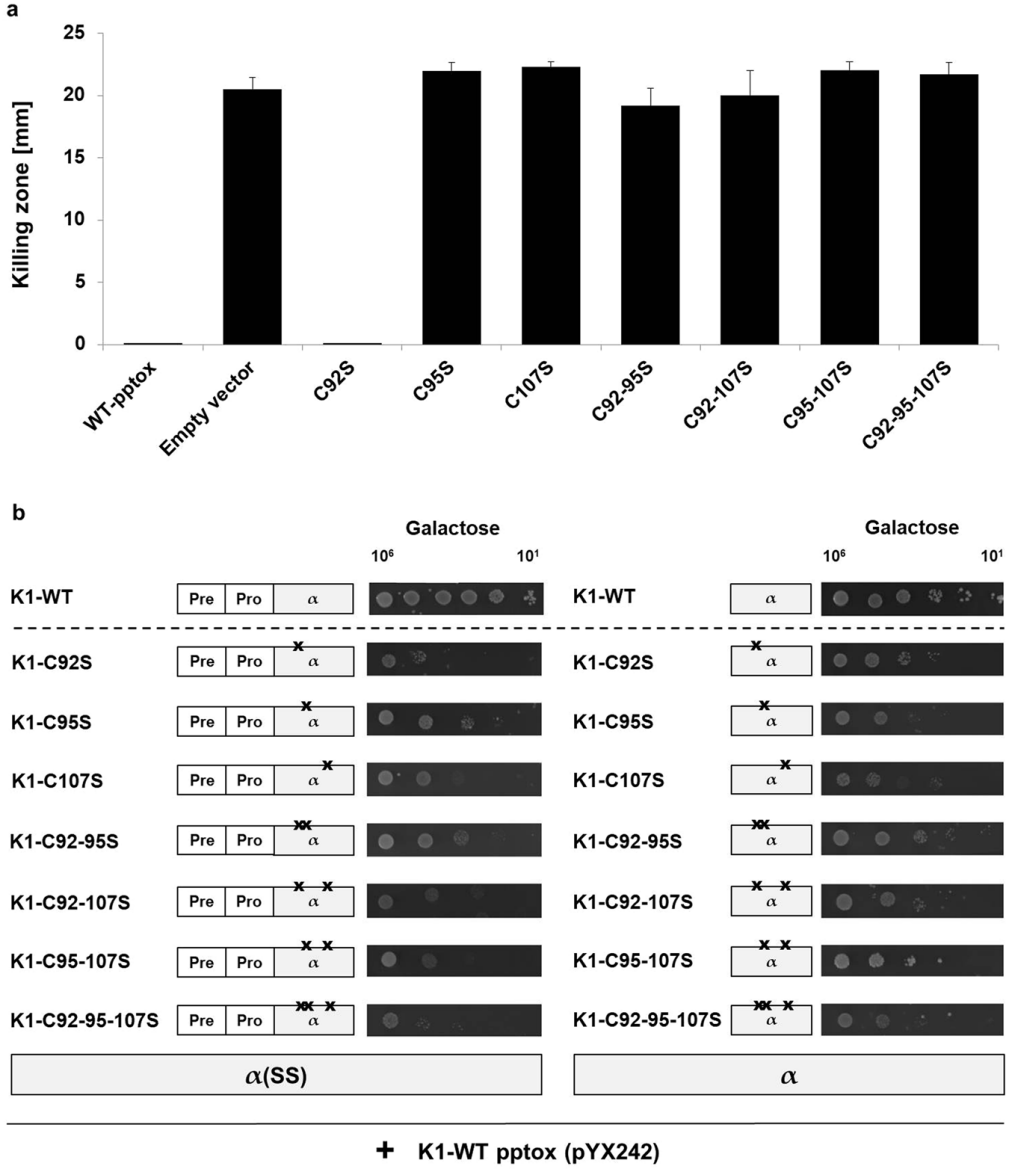
(**a**) Mutation of C95 and/or C107 results in loss of functional immunity. Yeast strain *S*. *cerevisiae* BY4742 was transformed with mutated K1-pptox derivatives and expression was induced by shifting the transformants in galactose-containing medium. 10^6^ cells were embedded into MBA (ura-galactose MBA, pH 4.7), and 1,000 AU K1 toxin concentrate were applied. Killing zones were measured after incubation of the plates for 3 d at 20 °C. Wild-type K1 pptox (WT-pptox) and empty vector were used as controls. (**b**) Cysteine mutations in K1 α subunit abolish the formation of immunity. Yeast cells of the *S*. *cerevisiae* strain BY4742 were co-transformed with wild-type K1 pptox and the indicated K1 lethal constructs either with an N-terminal prepro-region (α(SS), left) or without this region (α, right). 10^6^ to 10^1^ cells of each transformant were spotted onto galactose-containing agar plates (induced conditions); wild-type lethal constructs were used as control. Growth was analysed after 3 d at 30 °C, and one representative experiment is shown (n = 5).

Next, we analysed the ability of the cysteine-mutated toxin precursor variants to mediate immunity against externally applied K1 toxin. Interestingly, the substitution of cysteines C95 and C107 led to a complete loss of immunity resulting in a distinct zone of growth inhibition. Consequently, double mutants involving one of these cysteines and the respective triple mutant (C92-C95-C107S) were also not able to develop immunity against K1 toxin. Remarkably, the single mutation of C92 resulted in a phenotype comparable to the wildtype K1 pptox retaining full immunity (Fig. 3a). In case of the derivatives carrying cysteine substitutions in the β subunit, no alterations in toxin sensitivity were observed in comparison to the wildtype pptox control, and complete functional immunity was mediated (summary of all derivatives in Fig. 1c,d).

As already speculated in the literature, these data underline the involvement of the α subunit not only in the lethal effect but also in the mediation of proper immunity. Solely the single substitution of C92 with a serine residue produced a toxin precursor variant which was able to confer immunity against externally applied wild-type K1 toxin. In a prior study, a comprehensive mutational analysis of the K1 precursor was conducted where different sections and amino acids were mutated^24^. Besides many mutations disrupting α-helical structures in the toxic α subunit, the cysteines C92 and C312 were substituted with a tyrosine residue and a leucine residue, respectively. In case of the latter, the mutated toxin derivative was still immune against wild type K1 toxin and able to kill spheroplasts of sensitive cells; in contrast, the substitution of C92 with a tyrosine residue resulted in a loss of immunity pointing to sterically hindering conformational changes due to the phenolic group^24^. Our findings, therefore, not only support the proposed multi-functionality of α but also underline the importance of net charge and secondary structure of α for the mediation of immunity.

In line with this, we tested if the intracellular expression of mutated lethal constructs impairs the ability of the wild-type toxin precursor to mediate functional immunity in co-transformational experiments. Whereas co-expression of wild-type pptox with wild-type lethal α constructs conferred immunity against both α and α(SS) in serial dilution assays, co-transformation with mutated α constructs led to a significant decrease in cell viability for all tested derivatives. Most severe effects could be observed for α derivatives carrying the prepro-region, confirming ER import of the respective construct (Fig. 3b).

These data noticeably indicate that interaction of α with the pptox is necessary for the formation of immunity and that this mechanism involves the cysteines or at least specific secondary structures provided by these residues of both α and the pptox. Interestingly, the molecular mechanism of functional immunity seems to be not only completely independent of the toxic effect of the mutated K1 constructs but also the formation of a toxin dimer. Transformants expressing K1 pptox with a triple-mutated β subunit thereby excluding K1 heterodimer formation remained entirely immune against externally applied K1 toxin. This same phenotype could be observed for all analysed β mutants, and although none of these toxin derivatives was able to exert a toxic effect on whole cell level, toxin secretion could be verified for transformants expressing K1 derivates mutated in C248 and C312 and the corresponding double mutant C248-312. Additionally, lethal effects on sensitive spheroplasts could be detected for these mutant variants indicating not only disturbances in cell wall binding ability of these toxin derivatives but also correlating with previous findings demonstrating no involvement of β in the mediation of immunity^24^.

**Figure 4:**
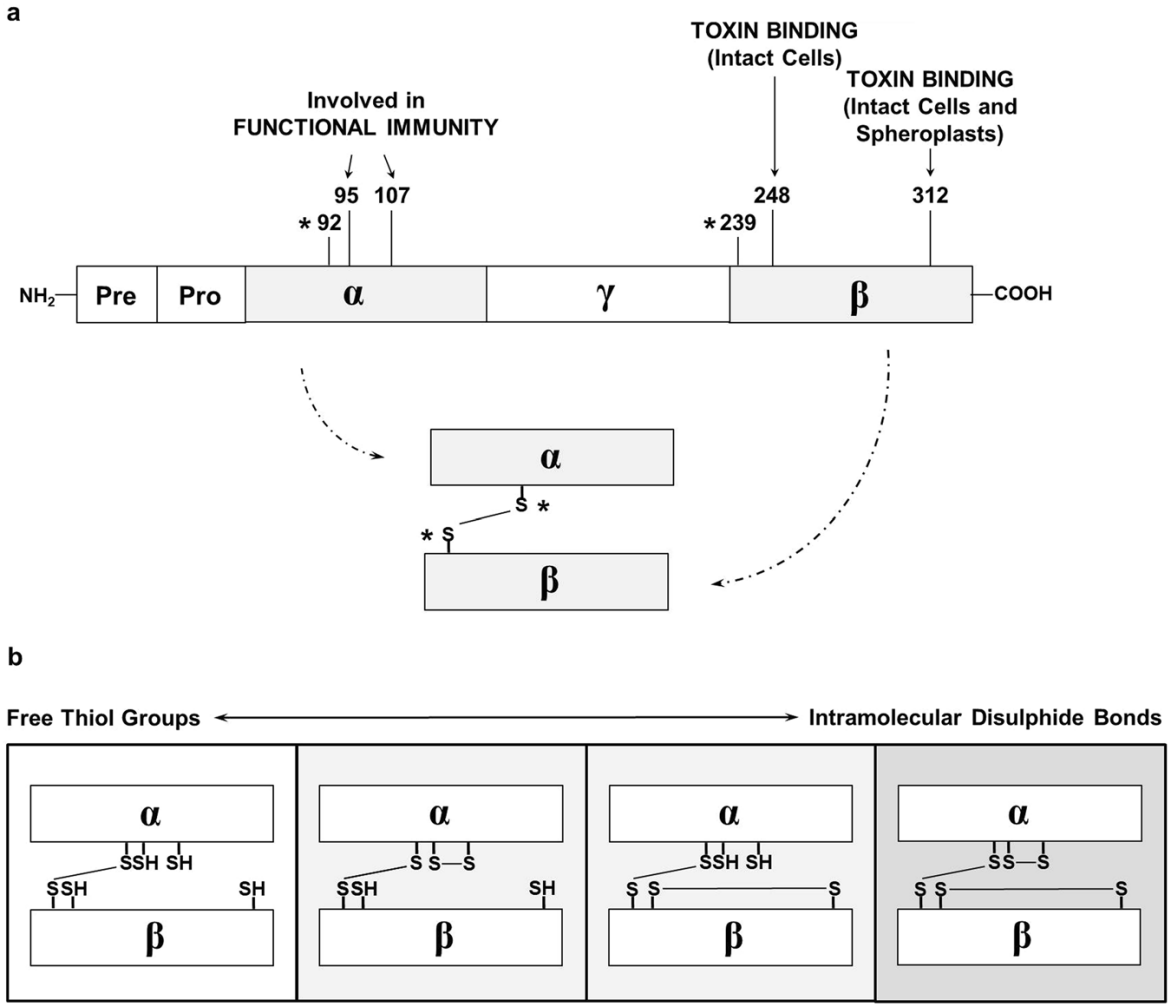
(**a**) Postulated functions of particular cysteine residues in K1 toxin. Results of cysteine substitutions within the K1 precursor molecule point to the formation of only one disulphide bond linking the major toxin subunits via C92-C239 (marked with*). Additionally, only mutations of C95 and C107 result in a complete loss of immunity indicating the mechanistic importance of these cysteines for this part of K1 toxin biology. Substitutions of C248 and C312 did not affect functional immunity but resulted in a loss of toxicity against intact cells (C248 and C312) and spheroplasts (C312) indicating critical functions within the toxin’s lethal mechanism. **(b**) Hypothetical disulphide arrangements within the heterodimeric K1 toxin. Cysteine residues not involved in linking α and β can either be present as free thiol groups or as part of intramolecular disulphide bonds. The possible rearrangements of the unbound thiol-groups are shown.

In summary, by systematic replacement of the cysteine residues within the K1 precursor, we propose the formation of only one disulphide bond in the mature heterodimeric toxin instead of three disulphide bonds stated in the literature for decades. Additionally, we demonstrate for the first time the importance of C95 and C107 for immunity against K1. We are now able to assign specific functions to the particular cysteine residues within the K1 pptox (Fig. 4a). Whereas C92 and C239 are involved in the development of the single disulphide bond between α and β, cysteines C95 and C107 are assumed to play a role in the formation of immunity against K1. Besides a direct involvement in the self-defence mechanism, these residues could potentially ensure the correct folding of the α subunit and thereby enabling the interaction of α with parts of the γ subunit. Although the importance of the latter in the mediation of functional immunity has already been shown, the exact role of the third toxin subunit γ needs further experimental validation^14^. In contrast, the remaining cysteines in β mediate toxin binding either to intact cells (C248) or intact cells and spheroplasts (C312). Based on these findings, four different redox-states of the remaining sulfuric residues in the heterodimeric toxin are possible, in which the cysteines that are not involved in the formation of the connecting disulphide bond might be able to cycle between their reduced and/or oxidised form (Fig. 4b).

However, future studies have to investigate if this switch between free thiol groups and intramolecular disulphide bonds actually occurs and which condition plays a physiological role in K1 biology. Additionally, the substitution of cysteine residues with the polar amino acid serine, even though being a close replacement concerning hydrophobicity, could lead to a misleading phenotype regarding protein function. Although no direct effect of the serine substitution on the K1-induced pore formation could be detected, any further impact, e. g. on tertiary structure, function, and stability of the toxin precursor, can not be entirely excluded. Especially as the toxic effect and immunity of K1 seem to be very prone for alterations in net charge and steric configuration, primarily when located in the toxic α subunit, additional studies could investigate potential effects of other amino acids. Substitution with the shorter non-polar amino acids alanine and valine, for example, could demonstrate potential effects of the side chain itself that are not directly linked to the formation of a disulphide bond. Analysis of the K1 heterodimer crystal structure could finally resolve its tertiary structure and verify the model proposed in this study. The knowledge of the toxin’s structural conformation could additionally be used for more refined site-directed substitutions, precisely targeting amino acids in specific positions within selected peptide regions. Nevertheless, our data provide unique valuable hints to elucidate the still unknown molecular mechanism/s leading to K1 toxicity as well as functional immunity and self-protection of toxin-producing cells.

## Methods

### Construction of K1 derivatives

*E*. *coli* strain TOP10 (F^−^ *mcr*A Δ(*mrr*-*hsd*RMS-*mcr*BC) Φ80*lac*ZΔM15 Δ*lac*X74 *rec*A1 *ara*D139 Δ(*ara leu*) 7697 *gal*U *gal*K *rps*L (StrR) *end*A1 *nup*G, Invitrogen) was used for cloning and amplification of all constructs. Cells were grown at 37 °C in LB medium (1% tryptone, 0.5% yeast extract, and 0.5% sodium chloride) supplemented with ampicillin for plasmid selection. Mutated K1 pptox constructs were synthesised as cDNA strings (GeneArt, Sigma) and adenine overhangs were added using *Taq* polymerase (Roche). K1 derivatives consisting of only the toxic α subunit (lethal constructs) were generated via PCR using the FastStart^™^ High Fidelity PCR System (Merck) and the respective primers (5′-K1 (5′-CTC GAG GAA TTC CAT ATG ACG AAG CCA ACC CAA GTA TTA GTT AGA TCC-3′), 5′-K1α (5′-CTC GAG GAA TTC CAT ATG GAA GCG CCG TGG TAT GAC AAG ATC TG-3′), 3′-K1α (5′-AGA TCT GTC GAC AAG CTT TTA AGC AAC GGT AGC GCC ATT AGG ATC TG-3′). Corresponding DNA fragments were cloned in the pYES2.1/V5-HIS-TOPO system (Invitrogen), and routinely sequenced. Subcloning was conducted into the yeast expression vector pYX242.

### *In vivo* expression of K1 derivatives

*Saccharomyces cerevisiae* strain BY4742 (MATα *his3*Δ*1 leu2*Δ*0 lys2*Δ*0 ura3*Δ*0*) was grown at 30 °C in YPD (1% yeast extract, 2% peptone, 2% glucose) and used for transformation via lithium acetate method^25^. Transformants were grown on suitable synthetic medium (SC) supplemented with appropriate amino acids and nucleotides (0.17% yeast nitrogen base, 0.5% ammonium sulfate, 2% glucose). Solid media were supplemented with 2% agar.

### Toxin production and concentration

K1 toxin was produced and concentrated as previously described using the killer strain T158c (MATα his4C-864)^22^. Toxicity of K1 concentrate was routinely checked via agar diffusion assay using SC-glucose methylene blue agar (MBA) with appropriate amino acids and nucleotide supplementation (0.5% ammonium sulfate, 1.92% citrate, 2% glucose, 0.17% yeast nitrogen base, 1.5% agar, methylene blue, pH 4.7). The diameter of K1-induced zone of growth inhibition (killing zone) is considered to reflect killer toxin activity further expressed in arbitrary units (AU). 1,000 AU correlate to a zone of growth inhibition of 10 mm in diameter against the *S*. *cerevisiae* strain BY4742.

### Methylene blue agar diffusion assay

Immunity against externally applied K1 toxin was determined with a standard agar diffusion assay using ura-galactose MBA (w/o uracil, 3% galactose, pH 4.7). Transformants were cultivated in ura d/o glucose medium and shifted to galactose-containing selective medium. After overnight incubation (30 °C, 200 rpm), 10^6^ cells of the respective transformant were embedded into the agar, and 1,000 AU K1 toxin concentrate was applicated into pre-cut holes. Plates were incubated at 20 °C for 3 d, and the diameter of the killing zones was measured indicating the loss of immunity.

### Analysis of toxin secretion

The secretion of K1 toxin was profiled via SDS-PAGE (non-reducing conditions) followed by Western Analysis. Transformants were shifted to galactose-containing medium to induce expression of the respective constructs and incubated for 3 d (20 °C, 220 rpm). After centrifugation, proteins of the supernatant were precipitated via trichloroacetic acid supplementation (14,000 rpm, 4 °C, 15 min). Colourimetric signal detection of the toxin dimer was conducted using a polyclonal primary antibody detecting the K1 dimer (α-K1, 1:1,000, rabbit) and a secondary antibody coupled with horseradish peroxidase (goat α-rabbit, 1:5,000, Roche).

### Biological activity of mutated K1 derivatives

Biological activity of secreted K1 toxin was analysed via MBA diffusion assay (SC-galactose, pH 4.7). In brief, 10^6^ cells of the sensitive strain BY4742 were embedded into the agar, and colonies of the respective transformants were streaked onto the agar (toxicity against intact cells). In case of spheroplasts, BY4742 cells were grown in YPD at 30 °C to late exponential phase (2–5 × 10^7^ cells/ml), harvested, and washed twice with sterile water. Cells were incubated in Tris/HCl-buffer (0.1 M Tris-HCl (pH 8.0), 5 mM DTT, 5 mM EDTA) for 30 min at 30 °C (150 rpm), and washed again. The pellet was resuspended in 1.2 M sorbitol (in 0.5 M Na_2_HPO_4_, pH 7.0), and Zymolyase was added to a final concentration of 500 µg/ml. After incubation for 2 h at 30 °C (100 rpm) cells were centrifuged (1,500 rpm, 5 min, RT), washed twice and resuspended in 1 ml 1.2 M Sorbitol. 100 µl of cell suspension was embedded into regeneration agar (1% yeast extract, 2% peptone, 3% galactose, 1.2 M sorbitol, 1.5% agar, methylene blue, pH 4.7). Colonies of the transformants were carefully streaked on the MBA surface. Killing zones were documented after incubation for 3–5 d at 20 °C.

### Characterisation of suicidal phenotype

Lethal effect of intracellular expressed K1 derivatives was analysed by performing serial dilution assays for each set of experiments as described previously^12^. 10^1^ to 10^6^ cells were spotted onto appropriate agar plates containing either glucose (non-induced control) or galactose (inducing conditions) as carbon source; plates were incubated for 3 d at 30 °C and cell growth was documented.

## Supplementary information

Dataset 1
