# Supplementary material for: Substitution of cysteines in the yeast viral killer toxin K1 precursor reveals novel insights in heterodimer formation and immunity
Source: Sci Rep. 2019 Sep 11;9:13127. doi: 10.1038/s41598-019-49621-z (PMC6739482; doi:10.1038/s41598-019-49621-z)
Supplement: Supplementary file 1 — Dataset 1 [file 41598_2019_49621_MOESM1_ESM.pdf]

Substitution of cysteines in the yeast viral killer toxin K1 precursor reveals novel insights in heterodimer formation and immunity

Stefanie Gier[1,2], Matthias Lermen[1,2], Manfred J. Schmitt[1,2], and Frank Breinig[1,2*]

[1]Molecular and Cell Biology

[2]Center of Human and Molecular Biology (ZHMB), Saarland University, 66123 Saarbrücken, Germany

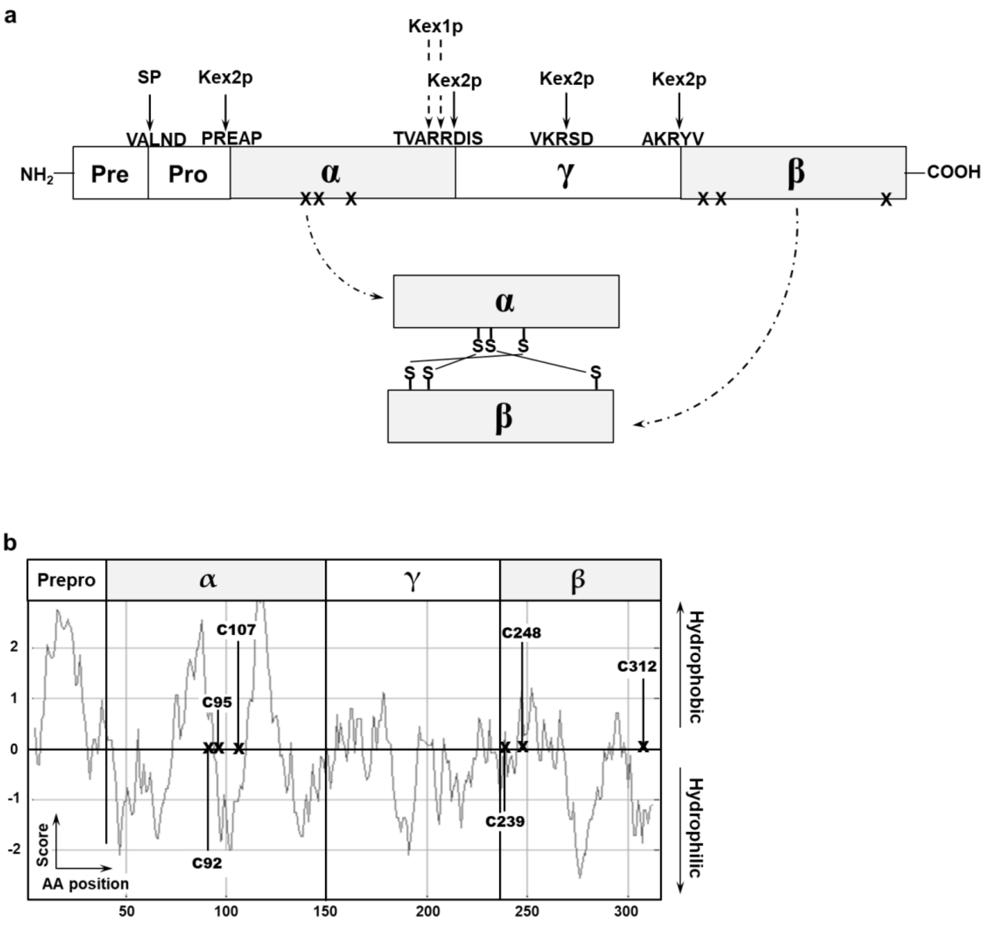

Supplementary Figure S1: (a) Schematic display of K1 precursor molecule. The published model postulates three disulphide bonds linking the major toxin subunits. Cleavage sites of signal peptidase complex (SP), Kex1p, and Kex2p are marked as arrows. **(b) Positions of the cysteine residues within the K1 precursor.** Depicted is a hydropathicity plot of K1 (Kyle & Doolittle; generated via Expasy). Cysteines C92, C95, C107 in α and C239, C248, C312 in β are highlighted as black crosses.

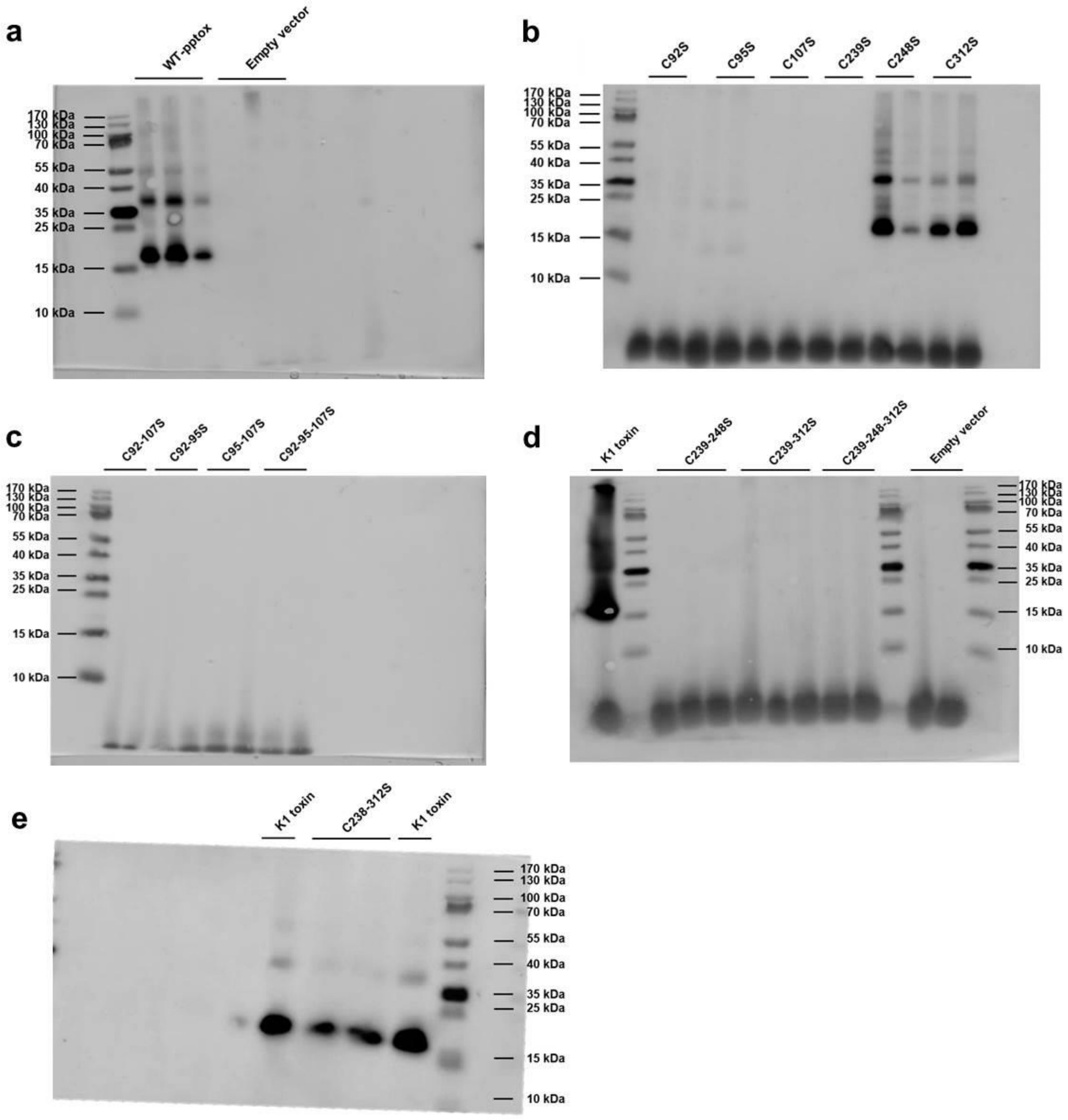

Supplementary Figure S2: Unmodified Western blot images displaying K1 heterodimer secretion of representative transformants. (a) Controls (WT-pptox, Empty Vector). **(b)** Single-mutated K1-pptox derivatives of α and β subunit. **(c)** Double and triple mutants of α subunit. **(d)** Double and triple mutants of β subunit (C239-248S, C239-312S, C239-248-312S) **(e)** Double mutant C248-248S. K1 Toxin: TCA-precipitated K1 toxin concentrate
